# Supplementary material for: Artificial intelligence and machine learning in mobile apps for mental health: A scoping review
Source: PLOS Digit Health. 2022 Aug 15;1(8):e0000079. doi: 10.1371/journal.pdig.0000079 (PMC9931284; doi:10.1371/journal.pdig.0000079)
Supplement: S2 Appendix — (DOCX) [file pdig.0000079.s002.docx]

## S2 Appendix. Search String

| **Database** | **Search String** | **Results** |
| --- | --- | --- |
| PubMed | ((Cell Phone OR Telemedicine OR Mobile Applications[MeSH Terms]) OR (Smartphone[Title/Abstract] OR mobile phone[Title/Abstract] OR mHealth[Title/Abstract] OR mobile health[Title/Abstract] OR "app"[Title/Abstract] OR "apps"[Title/Abstract] OR app-based[Title/Abstract] OR mobile[Title/Abstract] OR mobile application[Title/Abstract] OR mobile-based[Title/Abstract] OR phone-based[Title/Abstract] OR smartphone-based[Title/Abstract] OR medical informatics application[Title/Abstract] OR tablet[Title/Abstract] OR iPhone[Title/Abstract] OR android[Title/Abstract] OR iPad[Title/Abstract])) AND ((Mental Health OR Mental Health Services OR Mental Disorders OR Depression OR Anxiety Disorders OR Stress, Psychological OR Affect OR Mood Disorders OR Cognitive Behavioral Therapy OR Mental Health Recovery[MeSH Terms]) OR (Mental health[Title/Abstract] OR well-being[Title/Abstract] OR wellbeing[Title/Abstract] OR mental illness*[Title/Abstract] OR mental disorder*[Title/Abstract] OR psychological health[Title/Abstract] OR stress[Title/Abstract] OR anxiety[Title/Abstract] OR depression[Title/Abstract] OR mood[Title/Abstract] OR emotion[Title/Abstract] OR mental[Title/Abstract] OR wellness[Title/Abstract] OR distress[Title/Abstract] OR affective disorder*[Title/Abstract] OR psychotic disorder*[Title/Abstract] OR psychiatric disorder*[Title/Abstract] OR depressive[Title/Abstract] OR panic[Title/Abstract] OR psycho*[Title/Abstract] OR trauma*[Title/Abstract] OR insomnia[Title/Abstract] OR sleep problem[Title/Abstract] OR sleep disorder[Title/Abstract] OR self-harm[Title/Abstract] OR suicid*[Title/Abstract] OR cognitive behavioural therapy[Title/Abstract] OR cognitive behavioral therapy[Title/Abstract] OR CBT[Title/Abstract] OR mental health treatment*[Title/Abstract] OR mental health assessment*[Title/Abstract] OR therap*[Title/Abstract] OR mental health service*[Title/Abstract] OR psychotherapy[Title/Abstract])) AND ((Artificial Intelligence OR Natural Language Processing OR Machine Learning[MeSH Terms]) OR (Artificial intelligence[Title/Abstract] OR AI[Title/Abstract] OR conversational agent*[Title/Abstract] OR chatbot[Title/Abstract] OR chat bot[Title/Abstract] OR machine intelligence[Title/Abstract] OR intelligent support[Title/Abstract] OR machine learning[Title/Abstract] OR automated support[Title/Abstract] OR intelligent agent*[Title/Abstract] OR expert system[Title/Abstract] OR neural network[Title/Abstract] OR natural language processing[Title/Abstract] OR algorithm[Title/Abstract] OR deep learning[Title/Abstract])) | 1228    *(from 2014 to present)* |

### 
