# Supplementary material for: Artificial intelligence and machine learning in mobile apps for mental health: A scoping review
Source: PLOS Digit Health. 2022 Aug 15;1(8):e0000079. doi: 10.1371/journal.pdig.0000079 (PMC9931284; doi:10.1371/journal.pdig.0000079)
Supplement: S3 Appendix — (DOCX) [file pdig.0000079.s003.docx]

### S3 Appendix. Endnote search criteria

#### First screening

| **Pass^a^** | **Search string** | **# of references remaining** |
| --- | --- | --- |
| 1 | Any Field = artificial intelligence OR intelligent agent* OR conversational agent OR chatbot* OR chat bot* OR algorithm OR machine learning | 1022 |
| 2 | Any Field = smartphone app* OR phone app* OR mobile app* OR mHealth OR mobile application* OR mobile health app* OR mobile health OR iphone OR android OR tablet | 444 |
| 3 | Title = mental OR wellbeing OR well-being OR stress OR anxiety OR depress* OR support OR mood OR feeling* OR affect* | 221 |
| 4 | Title = study OR trial OR evalua* OR assess* OR RCT OR analy* OR feasib* OR pilot | 85 |
| 5 | Title = NOT (protocol OR symposium OR review OR meta-analysis OR conference OR rationale) | 68 |
| 6 | Abstract = (randomised control* OR randomized control* OR RCT OR cohort) | 14 |

^a^Each pass was conducted on the subset of studies retrieved in the previous pass.

#### Second screening

| **Pass^a^** | **Search string** | **# of references remaining** |
| --- | --- | --- |
| 1 | Any Field = artificial intelligence OR intelligent agent* OR conversational agent OR chatbot* OR chat bot* OR algorithm OR machine learning | 1022 |
| 2 | Any Field = smartphone app* OR phone app* OR mobile OR mHealth OR iphone OR android OR tablet | 688 |
| 3 | Title = mental OR wellbeing OR well-being OR stress OR anxiety OR depress* OR support OR mood OR feeling* OR affect* | 127 |
| 4 | Title = study OR trial OR evalua* OR assess* OR RCT OR analy* OR feasib* OR pilot | 54 |
| 5 | Title = NOT (protocol OR symposium OR review OR meta-analysis OR conference OR rationale) | 39 |

^a^Each pass was conducted on the subset of studies retrieved in the previous pass.
