# Supplementary material for: Artificial intelligence and machine learning in mobile apps for mental health: A scoping review
Source: PLOS Digit Health. 2022 Aug 15;1(8):e0000079. doi: 10.1371/journal.pdig.0000079 (PMC9931284; doi:10.1371/journal.pdig.0000079)
Supplement: S4 Appendix — (DOCX) [file pdig.0000079.s004.docx]

### S4 Appendix. Data Extraction

| **Reference** | **Characteristics** | **Strengths** | **Weaknesses** |
| --- | --- | --- | --- |
| Haines-Delmont et al. 2020 [[25]](https://paperpile.com/c/SeRWfa/FUu7) | -created a smartphone app called Strength Within Me, which was linked to Fitbit, Apple Health kit, and Facebook, to collect salient clinical information such as sleep behavior and mood, step frequency and count, and engagement patterns with the phone from a cohort of inpatients with acute mental health (n=66).  -aimed to apply machine learning in an acute mental health setting for suicide risk prediction | - results from this feasibility study indicate that, although not a perfect predictor, the KNN model is suitable for this study because it has shown the ability to separate users deemed at risk of suicide from the C-SSRS to those not deemed at risk at an average rate beyond just randomly guessing (ie, at an average rate 15% beyond randomly guessing the majority to be at low risk)  - Where the experiment excels in the data sources are diverse rather than strictly clinical and allow for natural extension to outpatient settings.  - Although in its early stages, research in this area suggests that using smartphones to enquire about suicidal behaviors can be a valuable approach and not a risk factor for increasing suicidal ideation | - limited by the short follow-up period of up to a week  - this study indicated that there are certain costs that limit the widespread adoption of health apps within mental health services (weather inpatient and community settings). These are related to access to smartphones, connectivity, updating, and maintenance of technology. (only a small number of inpatients had a smartphone) |
| Morrison et al. 2017 [[24]](https://paperpile.com/c/SeRWfa/qnEN) | - Intelligent sensor-driven machine learning models may improve the timeliness of notifications by adapting delivery to a user’s current context (e.g. location)  - exploratory mixed-methods study examined the potential impact of timing and frequency on notification response and usage of Healthy Mind, a smartphone-based stress management intervention.  - 77 participants were randomised to use one of three versions of Healthy Mind that provided: intelligent notifications; daily notifications within pre-defined time frames; or occasional notifications within pre-defined time frames. | - real world situation  - RCT | - Notification group had no meaningful effects on percentage of notifications viewed or usage of Healthy Mind  - No meaningful differences were indicated between the intelligent and non-intelligent groups.  - Our findings suggest that frequent notifications may encourage greater exposure to intervention content without deterring engagement, but adaptive tailoring of notification timing does not always enhance their use  - contrasting pattern of results observed in the current study highlights the need to evaluate emerging sensor-driven intervention models in a variety of contexts, particularly real-world use  - The sample size did not offer sufficient power to definitively test for between group differences.  - The effect sizes reported in this study should be considered tentative and no conclusions were drawn from small effects given that all confidence intervals crossed zero.  - design of this study did not permit us to examine the effect of notification group on perceived stress or other health-related outcomes |
| Sarda et al. 2019 [[26]](https://paperpile.com/c/SeRWfa/OidJ) | - analyze the association between smartphone-sensing parameters and symptoms of depression and to explore an approach to risk-stratify people with diabetes  - cross-sectional observational study was conducted on 47 participants with diabetes  -smartphone- sensing app passively collected data regarding activity, mobility, sleep, and communication from each participant. Self-reported symptoms of depression using a validated Patient Health Questionnaire-9 (PHQ-9) were collected once every 2 weeks  - The period of the study was originally 14 weeks and later extended to 20 weeks to collect sufficient smartphone-sensing data | - Participants with diabetes and self-reported symptoms of major depression were observed to show lower levels of social contact and lower activity levels during the day  - study shows promise in the use of predictive modeling for early detection of symptoms of depression in people with diabetes using smartphone-sensing information  - Using all the derived sensing variables, the extreme gradient boosting machine-learning classifier provided the best performance with an average cross-validation accuracy of 79.07% (95% CI 74%-84%) and test accuracy of 81.05% to classify symptoms of depression | - A noticeably high prevalence of self-reported symptoms of depression (63%) was observed in this study as compared with the 8% to 35% normally reported in other studies. This could be attributable to the single study site and the characteristics of the recruited participants  - The study was also limited by missing values in derived sensing variables: 1744 out of 2694 participant-day instances were removed from the dataset as they contained missing values in 1 or more of the derived sensing variables. Between large numbers of training instances available for modeling and avoidance of any bias being introduced in the dataset because of imputation of missing values in the derived sensing variables, a decision was taken in favor of the latter.  - Key limitations of the study included a single study site, small participant size, and the nonrandomized–based approach.  - The average of 2 consecutive PHQ-9 scores reported by a participant was assumed to be the depression symptom of the participant for the days and instances between the 2 screening time points. This could induce an error in outcome on days or instances where a participant was to exhibit a different mood or symptom. |
| Wahle et al. 2016 [[27]](https://paperpile.com/c/SeRWfa/pK10) | - The objective of this study is 2-fold, first to explore the detection of daily-life behavior based on sensor information to identify subjects with a clinically meaningful depression level, second to explore the potential of context sensitive intervention delivery to provide in-situ support for people with depressive symptoms.  - A total of 126 adults (age 20-57) were recruited to use the smartphone app Mobile Sensing and Support (MOSS), collecting context-sensitive sensor information and providing just-in-time interventions derived from cognitive behavior therapy.  - Real-time learning-systems were deployed to adapt to each subject’s preferences to optimize recommendations with respect to time, location, and personal preference.  - Machine-learning models used these features to infer behavior and context for PHQ-9 level prediction and tailored intervention delivery.  - Biweekly, participants were asked to complete a self-reported depression survey (PHQ-9) to track symptom progression. | - A total of 36 subjects used MOSS for ≥2 weeks. For subjects with clinical depression (PHQ-9≥11) at baseline and adherence ≥8 weeks (n=12), a significant drop in PHQ-9 was observed (*P*=.01). This group showed a negative trend between adherence and change in PHQ-9 scores (rho=−.498, *P*=.099).  - Proxies for social and physical behavior derived from smartphone sensor data was successfully deployed to deliver context-sensitive and personalized interventions to people with depressive symptoms.  - Subjects who used the app for an extended period of time showed significant reduction in self-reported symptom severity.  - Nonlinear classification models trained on features extracted from smartphone sensor data including Wifi, accelerometer, GPS, and phone use, demonstrated a proof of concept for the detection of depression superior to random classification | - The clinical study carried out is based on a nonrandomized, uncontrolled single-arm study design, which rules out the possibility to prove a direct causal link between symptom improvement and MOSS app use.  - in this first pilot we did not quantify the efficacy of the proposed recommendation algorithm, as this would involve detailed feedback from participants in order to judge appropriateness of context-related intervention recommendations. |
