# Supplementary material for: Artificial intelligence and machine learning in mobile apps for mental health: A scoping review
Source: PLOS Digit Health. 2022 Aug 15;1(8):e0000079. doi: 10.1371/journal.pdig.0000079 (PMC9931284; doi:10.1371/journal.pdig.0000079)
Supplement: Attachment — Submitted filename: PDIG-D-21-00085_review.docx [file pdig.0000079.s006.docx]

**General comments**

Mental health is increasingly recognized as a global public health issue. Mobile apps coupled with artificial intelligence and machine learning has become a promising tool to tackle the mental health issue on a large scale thanks to their accessibility and ability to process massive and diverse information. Yet the effectiveness is questioned. This review provides a landscape of current AI/ML-incorporated mobile apps for mental health, which is useful and timely. It also has a clear structure and is based on a reporting guideline to guide the writing.

In my opinions, I have few comments as below.

**Introduction**

The authors provided contrasting evidence of the effectiveness of mobile apps for supporting mental health. It would be useful to have a few explanations about the inconsistency and a summary of suggestions for how ‘further, high-quality research’ should be conducted ‘to provide evidence of effectiveness’. These suggestions would help the audience understand what has already been known as challenges in this kind of research. It could also elaborate further discussion later in the manuscript.

What has been known about AI/ML algorithms for mental health? Which variables/ data have been analysed?

**Methods**

The part is quite long in comparison to other parts of the manuscript. Table 2 can be put in the appendix.

In the PICO table, the part ‘Study types’ stated RCTs and cohort studies. However, there is a cross-section study in the results. Suggests changing to ‘RCTs and observational studies.

Were there any restrictions in terms of publishing time and language?

As mental health is an umbrella term including a wide range of mental health conditions, the authors should mention their definition for the one used in this review.

For the data extraction, only the age of participants was extracted for each study. Since the overall aim of the review is to explore the use of AI/ML in mobile apps, it is important to have details about who using these apps, whether or not it is similar to the population mentioned in the PICO table or the real world etc. Also, the settings/countries of the participants in the reviewed studies.

**Results**

Is there any information about the validated and reliable measures through the apps (sensor data, questionnaires, etc.)

In the study findings part, further information should be added:

-	What are the study participant characteristics (maybe demographics, mental health conditions, etc.)? Without this information, it is hard to discuss the representativeness in the discussion part.

-	What are the outcomes measured in each study?

-	What are the strengths and weaknesses of the study reviewed?

**Discussion**

The discussion is a bit descriptive. The strengths and weaknesses of AI/ML in apps are likely to be on the results in my view.

Critical appraisals should be made in terms of how the findings of this review align or contrast with the current knowledge. Albeit the scope of the review is the use of AI/ML-enabled mobile apps for mental health, the discussion should be explained into how the findings would fit or unfit with the current research landscape of digital health.

As there are different data types collected for each study, do the authors have any comments about the standardization and integration of data?

I am particularly interested in health equity which might be out of the scope for this review, but any comments can be drawn from this review (representativeness, generalization of the study findings, etc.)

**Conclusion and future research**

Are there any specific recommendations based on what we found in this review? The phrase ‘high-quality RCTs’ is a bit vague and not informative, unfortunately.
