# Supplementary material for: Artificial intelligence and machine learning in mobile apps for mental health: A scoping review
Source: PLOS Digit Health. 2022 Aug 15;1(8):e0000079. doi: 10.1371/journal.pdig.0000079 (PMC9931284; doi:10.1371/journal.pdig.0000079)
Supplement: Attachment — Submitted filename: Response to Reviewers (mental health scoping rev).docx [file pdig.0000079.s007.docx]

| PLOS Digital Health |  |  |
| --- | --- | --- |

11 March 2022

Dear Bridianne,

Thank you for your consideration of our manuscript “Artificial intelligence and machine learning in mobile apps for mental health: A scoping review of randomised controlled trials and cohort studies” for publication in PLOS Digital Health. We have made amendments to the manuscript reflecting the valuable review comments forwarded and feel the paper has been well enhanced to be re-presented for publication. Please let us know if there is anything we can further do to clarify or extend our paper.

With kind regards,

Edward Meinert

Enclosures:

1. Chart mapping peer review feedback

| **ID** | **Reviewer** | **Reviewer Feedback** | **Author response** | **Action taken** |
| --- | --- | --- | --- | --- |
| 1 | Editor | Thank you for your patience in the review of your recent submission to PLOS Digital Health for the manuscript titled "Artificial intelligence and machine learning in mobile apps for mental health: A scoping review of randomised controlled trials and cohort studies". I apologise that this may have taken longer than you expected.  We were able to secure four reviewers for your manuscript. Together, we agree that the manuscript requires major revision before we can accept for publication. A major concern is the small number of included studies, which may have been impacted by your limited search strategy. I would like to give you the opportunity to respond to the reviewers' comments prior to making a final decision on the manuscript.  Please find attached the reviewers comments.  I look forward to reading your response. | We thank the editor for their time and consideration in the review of our manuscript. The raised issues have been addressed in view of the peer review feedback provided. | N/A |
| 2 | Reviewer 1 | Thank you for conducting this scoping review. Please find below my comments and suggested edits. | We thank the reviewer for their time and consideration in the review of our manuscript. The raised issues have been addressed in view of the peer review feedback provided. | N/A |
| 3 | Reviewer 1 | Although included as a study limitation, please justify why only PubMed was searched for this scoping review in the methodology section. | PubMed was selected because it provides a good synthesis of biomedical literature. The PubMed database includes over 33 million citations, with around 30,000 records included in the PubMed journal list. The authors chose to focus on one database because of time limitations and determined the PubMed would provide good coverage of the literature. A recent article examining PubMed coverage found that it covered around 80% of studies included in Cochrane reviews. <https://doi.org/10.1016/j.jclinepi.2019.04.015> | N/A |
| 4 | Reviewer 1 | Please also justify the timeframe for the search (from 2014). Both 1 & 2 may impact the number of studies included in the review and potentially alter the findings. | Apple, Google, Amazon, and Microsoft introduced AI-based voice assistants from 2010-2014 (Siri, Google Now, Alexa, and Cortana), marking the early introduction of AI to mobile phones. Preliminary searches of PubMed also identified an exponential increase in publications starting in 2014. For these reasons, we limited the search results to 2014. We have added text in the methods to clarify this justification in the paper. | Sentences and citation added |
| 5 | Reviewer 1 | Please justify why quality assessment was not conducted for the included studies | We appreciate the reviewer indicated that this was not clear to them and have added an explanation of our decision not to conduct a critical appraisal of quality in the methods section. We followed the PRISMA-ScR guidance for conducting scoping reviews, and determined in alignment with that guidance that a critical appraisal was not needed to achieve the main aim of providing an overview of the incorporation of AI and ML in mental health apps. However, we agree that this will be an important area for future systematic reviews. | Sentences added |
| 6 | Reviewer 1 | Typo on page 8, second paragraph, ‘assessed apps aiming to identify…’ | We thank the reviewer for pointing this out and have addressed in text. | Typo fixed |
| 7 | Reviewer 1 | Typo on page 10, second paragraph, ‘found no difference’ | We thank the reviewer for pointing this out and have addressed in text. | Typo fixed |
| 8 | Reviewer 1 | Why did the two reviewers collaborate instead of working independently? | Two reviewers screened the articles separately. Full text inclusion and data analysis were conducted by one reviewer and then validated by a second. The second reviewer also added to the data analysis, based on their speciality of research. This is why the data analysis was done collaboratively, so that the different authors could analyse the aspects of the studies that they had more experience evaluating. | Sentence added in text to clarify |
| 9 | Reviewer 2 | This paper presents a scoping review of studies of the impact of AI and machine learning in mental health apps. Noting the growing prevalence and availability of mental health apps, the authors used solid methodological approaches - namely PRISMA-ScR and PICOS – to structure a sysetmatic review. Four papers were identified and key aspects of those papers were exracted and compared.  This paper presents a well-thought out and a (mostly) methodologically sound examination of a useful and important question.  However, the review as presented provides minimal insight. | We thank the reviewer for their time and consideration in the review of our manuscript. The raised issues have been addressed in view of the peer review feedback provided. | N/A |
| 10 | Reviewer 2 | The immediate reason lies in the small size of the result set. With only four papers, it's impossible to identify any meaningul trends or pattners.  Given the small number of papers included, it would seem imminently possible to spend a few sentences on each, leading to a potentially richer comparison. For example, I would be interested in learning about similarities and differences between the two depression apps. Similarly, the findings description omits important information about exactly what the end points where and how they were assessed. With four studies, these details could be discussed explicitly. | We agree that the insight of the paper is limited by the small number of included studies, but given our pre-determined inclusion and exclusion criteria, they were the only four studies that were eligible for examination. This in itself provides a useful observation about the lack of rigorous evaluations of AI/ML technologies in mental health apps. We have, however, added more details about the apps included in the review, as the reviewer has suggested. | Further app description added to results |
| 11 | Reviewer 2 | The small number of pappers raises the possibility that the area is still to under-developed, or the criteria are simply too small for a scoping review. With over 1000 papers in the original set and only four that met eligibility criteria, I am left wondering about the 997 that were excluded during the final filter.  Although a scoping review of this broader set, or some relevant subset thereof, would almost certainly be an entirely different paper, the current paper would likely be strengthened by some discussion of the broader landscape. | The authors agree that another review examining the development of AI/ML-enabled mobile apps for mental health would provide further useful insights into the field, but we also agree that this would be a different paper, as our scope was clearly limited to evaluations of those mobile apps. The lack of evaluations of such apps indicates that this will be an important area to observe in future, to determine the reasons for the lack; whether the AI/ML mental health apps are simply in too early stages at the moment and evaluations are upcoming, or whether there is a failure of companies to rigorously evaluate tools that are being made available to the public. | N/A |
| 12 | Reviewer 3 | How you can integrate the RCT  and cohort study design in your research finding? how could you analysis ? | The authors are not entirely sure what the reviewer means, but as there were so few studies included, all studies have been analysed together. When more evaluations have been published, it might be possible to conduct a meta-analysis of outcomes, although the variety of different areas of focus and aims of the mental health apps could make this challenging. | N/A |
| 13 | Reviewer 3 | How you limit Variables  for Artificial intelligence and machine learning in mobile apps? is feasible? | The authors are also not sure what the reviewer means with this comment; one of the benefits of using AI/ML is their ability to collate and analyse many different variables (for instance, as papers [25] and [26] did with risk stratification algorithms for depression and suicide risk). If the reviewer means how to limit variables to reduce the scope of the review and enable more in depth evaluation, this could be done by limiting future reviews to a specific type of AI or ML technique, to enable more direct comparisons. | N/A |
| 14 | Reviewer 4 | The review was rigorously conducted and structured according to an international reporting guideline. There is no statistical analysis in this review due to the nature of this type of study. The review helps inform the overall landscape of research about the AI/ML-enabled mobile apps for mental health which has a great relevance to the current needs. The manuscript is professionally written which makes the manuscript a pleasure to read. | We thank the reviewer for their time and consideration in the review of our manuscript. | N/A |
| 15 | Attached review | **General comments**  Mental health is increasingly recognized as a global public health issue. Mobile apps coupled with artificial intelligence and machine learning has become a promising tool to tackle the mental health issue on a large scale thanks to their accessibility and ability to process massive and diverse information. Yet the effectiveness is questioned. This review provides a landscape of current AI/ML-incorporated mobile apps for mental health, which is useful and timely. It also has a clear structure and is based on a reporting guideline to guide the writing.  In my opinions, I have few comments as below. | We thank the reviewer for their time and consideration in the review of our manuscript. The raised issues have been addressed in view of the peer review feedback provided. | N/A |
| 16 | Attached review | **Introduction**  The authors provided contrasting evidence of the effectiveness of mobile apps for supporting mental health. It would be useful to have a few explanations about the inconsistency and a summary of suggestions for how ‘further, high-quality research’ should be conducted ‘to provide evidence of effectiveness’. These suggestions would help the audience understand what has already been known as challenges in this kind of research. It could also elaborate further discussion later in the manuscript. | To address the reviewer’s comment that the introduction and issue of research quality was slightly unclear, the authors have added examples of some of the methodological issues identified in previous systematic reviews of mental health apps. We have also added a recommendation for well-accepted design and reporting guidelines that can be used to improve the reporting and validity of RCTs. | Sentences and citations added |
| 17 | Attached review | What has been known about AI/ML algorithms for mental health? Which variables/ data have been analysed? | Determining the answer to this question was among the aims of our scoping review, and the data we extracted are described in our results. Examining the use of AI/ML in mental health beyond the context of mobile apps was out of the scope of our review, although the authors agree that this would be an interesting area for examination. | N/A |
| 18 | Attached review | **Methods**  The part is quite long in comparison to other parts of the manuscript. Table 2 can be put in the appendix. | The full search string is already included as an appendix, so we have removed Table 2 but not included it as an additional appendix. | Table removed |
| 19 | Attached review | In the PICO table, the part ‘Study types’ stated RCTs and cohort studies. However, there is a cross-section study in the results. Suggests changing to ‘RCTs and observational studies.  Were there any restrictions in terms of publishing time and language? | The authors appreciate the reviewer pointing out this discrepancy; we have changed the PICOS table in accordance with the reviewer’s suggestion. | Table 1 edited |
| 20 | Attached review | As mental health is an umbrella term including a wide range of mental health conditions, the authors should mention their definition for the one used in this review. | We thank the reviewer for identifying that this was not clear, we have described our inclusion criteria for mental health support in greater detail. | Sentences added |
| 21 | Attached review | For the data extraction, only the age of participants was extracted for each study. Since the overall aim of the review is to explore the use of AI/ML in mobile apps, it is important to have details about who using these apps, whether or not it is similar to the population mentioned in the PICO table or the real world etc. Also, the settings/countries of the participants in the reviewed studies. | To address this and the following two comments, the authors have added a table providing more detail about the data extracted about the study characteristics, including the initial and analysed sample sizes, demographic characteristics of the sample, the study setting and country. | Table added |
| 22 | Attached review | **Results**  Is there any information about the validated and reliable measures through the apps (sensor data, questionnaires, etc.) | The authors have included reference to validated questionnaires used in the studies in the newly added Table 2 where applicable as suggested by the reviewer. | Text added |
| 23 | Attached review | In the study findings part, further information should be added:  - What are the study participant characteristics (maybe demographics, mental health conditions, etc.)? Without this information, it is hard to discuss the representativeness in the discussion part.  - What are the outcomes measured in each study?  - What are the strengths and weaknesses of the study reviewed? | As described in the response to comment 21, the authors have added a table providing additional detail to the results section. In addition to the study and participant characteristics described in that response, the authors also included the mental health condition(s) that the study focused on and the outcomes measured in the table. We have moved the discussion of the strengths and weaknesses of the studies to the results section as suggested in comment 24. | Table added |
| 24 | Attached review | **Discussion**  The discussion is a bit descriptive. The strengths and weaknesses of AI/ML in apps are likely to be on the results in my view.  Critical appraisals should be made in terms of how the findings of this review align or contrast with the current knowledge. Albeit the scope of the review is the use of AI/ML-enabled mobile apps for mental health, the discussion should be explained into how the findings would fit or unfit with the current research landscape of digital health. | We thank the reviewer for the suggestion and agree that the strengths and weaknesses of AI/ML in app studies fits into the results section. We have elaborated on how the results of this review align with the literature in the conclusion. | Paragraphs moved to results; sentences and citations added to conclusion |
| 25 | Attached review | As there are different data types collected for each study, do the authors have any comments about the standardization and integration of data? | The different types of studies and data collected – as well as the paucity of studies eligible for inclusion – meant that we couldn’t do a meta-analysis, so we did not feel that the standardisation and integration of data in depth was within the scope of this review. However, we agree that this will be an important area to consider for future studies, which may need to limit scope to particular types of studies or artificial intelligence technologies to ensure that meta-analyses can be conducted. | N/A |
| 26 | Attached review | I am particularly interested in health equity which might be out of the scope for this review, but any comments can be drawn from this review (representativeness, generalization of the study findings, etc.) | The authors are also interested in health equity; particularly as a significant potential benefit of digital health technology is its (often unrealized) potential to improve accessibility to healthcare services. However, the reviewer is correct that that was out of the scope of this review, as the studies were in relatively early stages of evaluation with small sample sizes. This will be an important area for future research, and we would welcome a review on the topic. | N/A |
| 27 | Attached review | **Conclusion and future research**  Are there any specific recommendations based on what we found in this review? The phrase ‘high-quality RCTs’ is a bit vague and not informative, unfortunately. | We thank the reviewer for raising this query, and we have added a sentence in the conclusion to clarify specific recommendations for future studies. | Sentence added |
