# Supplementary material for: Artificial intelligence and machine learning in mobile apps for mental health: A scoping review
Source: PLOS Digit Health. 2022 Aug 15;1(8):e0000079. doi: 10.1371/journal.pdig.0000079 (PMC9931284; doi:10.1371/journal.pdig.0000079)
Supplement: Attachment — Submitted filename: Response to Reviewers (mental health scoping rev) - 2.docx [file pdig.0000079.s008.docx]

| PLOS Digital Health |  |  |
| --- | --- | --- |

15 June 2022

Dear Bridianne,

Thank you for your consideration of our manuscript “Artificial intelligence and machine learning in mobile apps for mental health: A scoping review of randomised controlled trials and cohort studies” for publication in PLOS Digital Health. We have made amendments to the manuscript reflecting the valuable review comments forwarded and feel the paper has been well enhanced to be re-presented for publication. Please let us know if there is anything we can further do to clarify or extend our paper.

With kind regards,

Edward Meinert

Enclosures:

1. Chart mapping peer review feedback

| **ID** | **Reviewer** | **Reviewer Feedback** | **Author response** | **Action taken** |
| --- | --- | --- | --- | --- |
| 1 | Editor | 1. Please update the completed 'Competing Interests' statement. Please declare all competing interests beginning with the statement “I have read the journal's policy and the authors of this manuscript have the following competing interests:”. | The authors thank the editor for pointing out this oversight; we have added the journal’s standard beginning text to the competing interests statement. | Text added |
| 2 | Editor | Both reviewers raised concerns regarding the fact that only 4 studies were ultimately included in this scoping review. Looking at this manuscript, I also find that concerning, for a couple of reasons: 1. An important RCT seems to be missing, by Fitzpatrick 2017 (DOI: 10.2196/mental.7785) and it is not clear to me why that might be the reason from looking at the eligibility criteria. 2. The eligibility criteria are not specific enough and the authors do not define what they mean by "Mobile apps using artificial intelligence (AI) and machine learning (ML) techniques to address issues relating to mental health". How did you define AI? By including any rule-based approach it is simply impossible that only 4 articles would be included. | We thank the editor for their time and consideration in the review of our manuscript. The raised issues have been addressed in view of the peer review feedback provided.  We appreciate the reviewer’s recognition of the importance of knowing where there are research gaps. Based on this feedback, and the identified paper, we reviewed and revised our screening terms, redid the screening, included and extracted data from 13 additional papers, and redrafted the results and conclusions. | Update of the screening and data extraction, revision of text |
| 3 | Editor | 3. I believe the conclusion are overstating the results: "The studies included in the review demonstrated the feasibility of incorporating AI/ML into mental health apps." | We thank the editor for pointing out that this might be an overstatement, based on the evidence. We have revised the discussion and this sentence in particular based on the inclusion of additional papers. | Text changed |
| 4 | Editor | 4. There is an apparent mismatch between eligible and included study designs, as a crossectional study seems to be 1 of the 4 studies included? In characterising study designs, authors should refrain from using the terms in the original papers and rather use a standardised approach (i.e. "cohort study" and "clinical pilot study" are not very informative terms). | We thank the editor for identifying this mismatch, we have revised the description of included studies within the PICOS framework table. | Table changed, inclusion criteria updated |
| 5 | Reviewer 2 | Comments to the author: (No Response) | We thank the reviewer for their time and consideration in the review of our manuscript. The raised issues have been addressed in view of the peer review feedback provided. | N/A |
| 6 | Reviewer 2 | Meet PLOS DH criteria: Yes |  |  |
| 7 | Reviewer 2 | Statistical analysis: N/A |  |  |
| 8 | Reviewer 2 | Data availability: Yes |  |  |
| 9 | Reviewer 2 | Intelligible English: Yes |  |  |
| 10 | Reviewer 2 | Thanks you for acknowledging the limited insight available from a scoping review of only four papers. I appreciate the suggestion that the limited number of papers is in itself a useful insight, but this does leave me to question the overall contribution of the paper. | We appreciate the reviewer’s recognition of the importance of knowing where there are research gaps. Based on peer review feedback, we revised the screening criteria and redid the screening, included and extracted data from 13 additional papers, and redrafted the results and conclusions. | Update of the screening and data extraction, revision of text |
| 11 | Reviewer 4 | All comments have been addressed | We thank the reviewer for their time and consideration in the review of our manuscript. The raised issues have been addressed in view of the peer review feedback provided. | N/A |
| 12 | Reviewer 4 | Meet PLOS DH criteria: Yes |  |  |
| 13 | Reviewer 4 | Statistical analysis: N/A |  |  |
| 14 | Reviewer 4 | Data availability: Yes |  |  |
| 15 | Reviewer 4 | Intelligible English: Yes |  |  |
| 16 | Reviewer 4 | The authors have made great efforts in addressing the comments and clarifying the questions from the reviewers. The revised manuscript therefore becomes clearer and more informative. Albeit only four papers were included in the review, the manuscript provides a nice summary of the current research landscape of AI-enabled mobile apps for mental health. Personally, it is intriguing to see such a small number of research apps amongst a huge number of commercialised apps for mental health out there. | We appreciate the reviewer’s recognition of the importance of knowing where there are research gaps. Based on peer review feedback, we revised the screening criteria and redid the screening, included and extracted data from 13 additional papers, and redrafted the results and conclusions. | Update of the screening and data extraction, revision of text |
